# Mitochondrial Genome Evolution: The Influence of Partitioning, Calibration, and Gene Heterogeneity on Pleurodontan Substitution Rates

**DOI:** 10.1007/s00239-026-10324-5

**Published:** 2026-05-30

**Authors:** Matheus M. A. Salles, Fabricius M. C. B. Domingos

**Affiliations:** Departamento de Zoologia, Setor de Ciências Biológicas, Programa de Pós-Graduação em Zoologia, Universidade Federal do Paraná, Centro Politécnico, Avenida Cel. Francisco H Santos, Jardim das Américas, Curitiba, PR 81531-980 Brazil

**Keywords:** Divergence time, Mitogenome, Molecular evolution, Phylogenomics, Squamata

## Abstract

Substitution rate estimates are central to evolutionary biology, underpinning divergence-time inference and a wide range of macroevolutionary analyses. Mitochondrial DNA (mtDNA) rates are widely used for this purpose, yet they are often derived from a limited set of genes, closely related taxa, or a small number of model organisms. Here, we use nearly complete mitogenomes from 27 pleurodontan species (Squamata: Pleurodonta) to estimate substitution rates across the mitochondrial genome, explicitly evaluating the effects of data partitioning, calibration strategies, and model specification. Bayesian analyses revealed pronounced heterogeneity in substitution rates among codon positions and between coding and non-coding regions. Estimated rates ranged from approximately 0.004 to 0.02 substitutions per site per million years, consistent with previous lineage-specific estimates. Commonly used rates closely matched those estimated for third codon positions and for analyses based on combined partitions, suggesting that widely adopted values may primarily reflect signals from faster-evolving sites or aggregated partitioning schemes. Calibrated analyses generally yielded lower substitution rate estimates with reduced variance relative to non-calibrated analyses. However, substantial overlap in 95% highest posterior density intervals indicates limited evidence for systematic differences between these approaches, suggesting that much of the relative rate structure is already captured by the molecular data under a partitioned relaxed-clock framework. Comparisons between alternative partitioning strategies further showed that, although data-driven schemes recover broad patterns of rate variation, they may do so at the cost of reduced parameter resolution, particularly in the absence of calibration. Together, these results highlight that substitution rate estimates are sensitive to partitioning and modeling choices, and that model complexity should be evaluated in terms of parameter resolution and data informativeness rather than parameter count alone. By providing partition-specific rate estimates across the mitogenome, this study offers a robust empirical framework for improving molecular dating and evolutionary inference in squamates and other non-model systems.

**Supplementary Information:**

The online version contains supplementary material available at 10.1007/s00239-026-10324-5.

## Introduction

Genomic datasets are essential for addressing complex questions in modern evolutionary biology. In this context, substitution rate estimates are a cornerstone, providing critical insights into molecular evolution and serving as a foundation for various applications. For instance, in the absence of fossils or other secondary calibration points, substitution rates often represent the main available data for estimating divergence times (e.g., Ho 2007; Arcones et al. 2021). Mitochondrial DNA (mtDNA), in particular, has long been used for this purpose, mainly due to its relatively stable coding function, high mutation rates, small effective population size, matrilineal inheritance, and relatively fast coalescent times (Avise et al. 1987; Ballard and Rand 2005). Besides, mitochondrial proteins play a critical role in the oxidative phosphorylation pathway and exhibit functional conservation across different metazoan lineages (Gray et al. 1999; Broughton and Reneau 2006). Consequently, the accuracy of mitochondrial substitution rate estimates is fundamental to advancing evolutionary biology.

Substitution rates vary considerably across the mitochondrial genome and among different taxonomic groups. Empirical studies have revealed substantial variation among different mitochondrial genes (Williams and Hurst 2002; Sloan et al. 2009; Pons et al. 2010; Duchêne et al. 2011; Zhu et al. 2014; Borges et al. 2025) as well as across lineages (Parkinson et al. 2005; Bininda-Emonds 2007; Mower et al. 2007; Nabholz et al. 2008; Welch et al. 2008; Eo & DeWoody 2010; Yan et al. 2021). Importantly, many studies have historically relied on a limited fraction of the mitogenome—primarily cytochrome b, cytochrome c oxidase I, II, and III, and the 12S and 16S ribosomal RNAs (Johns and Avise 1998; Hebert et al. 2003; Roe & Sperling 2007; Patwardhan et al. 2014)—and have been based on a few model organisms, typically at the intraspecific level or between closely related species (Avise et al. 1987; Ballard and Whitlock 2004; Funk and Omland 2003; Ballard and Rand 2005; Rubinoff and Holland 2005).

Consequently, despite their widespread use, molecular clock approaches based on mtDNA have important practical limitations. Overlooking those variations can introduce substantial biases in substitution rate estimates, posing challenges for accurate evolutionary inference. This is particularly concerning in deep-level phylogenies, where errors in phylogenetic inference tend to amplify with increasing branch length (Buckley 2002; Lemmon et al. 2009). To mitigate this problem, some studies have attempted to calibrate molecular rates using complete (or nearly complete) mitogenomes, across different groups (Pons et al. 2010; Park et al. 2012; Plazzi et al. 2016; Mackiewicz et al. 2022). Such level of resolution is crucial, as accurate divergence time estimates rely on the precision and accuracy of calibration points and the rates applied to each marker and lineage under investigation (Mello and Schrago 2014; Zheng and Wiens 2015; Ritchie et al. 2017; Smith et al. 2018). Also, effective calibrations help to counteract errors arising from clock model misspecification (Duchêne et al. 2014). At the same time, increasing model realism through more complex analytical strategies (such as fine-scale partitioning schemes, flexible substitution models, and relaxed-clock frameworks) introduce additional parameters that may affect parameter identifiability and model behavior (Rannala 2002). In Bayesian phylogenetics, these trade-offs between model complexity and statistical power remain an important methodological consideration, particularly when estimating substitution rates across heterogeneous genomic regions. Therefore, evaluating how different partitioning strategies and model configurations influence empirical rate estimates is essential to ensure robust and biologically meaningful inferences.

Squamates (lizards, snakes, and amphisbaenians; Order Squamata) form a globally distributed clade of reptiles comprising approximately 11,000 extant species (Simões and Pyron 2021; Uetz et al. 2025), making them one of the most diverse vertebrate orders (Uetz et al. 2021). Despite recent advancements in next-generation sequencing, squamates remain underrepresented in genomic research compared to mammals and birds (Feng et al. 2020; Genereux et al. 2020; Gable et al. 2023). This limited genomic data availability hinders a comprehensive understanding of key evolutionary parameters within the group, including substitution rates. In particular, the Pleurodonta clade (the main focus of this study) encompasses a wide range of taxa predominantly distributed throughout the New World, with desert iguanas, horned, spiny, and collared lizards dominating many modern squamate faunas in North and South America (Pianka and Vitt 2003; Losos 2011; Avila et al. 2013; Carvalho et al. 2013). Although Pleurodontan evolutionary history is marked by multiple adaptive radiations in response to varied ecological pressures (Blankers et al. 2013; Alencar et al. 2024), mitochondrial evolutionary parameters remain scarce for the group. Commonly used substitution rate values broadly range from 0.005 to 0.02 substitutions per site per lineage per million years (subs/site/MY), depending on the gene (e.g., Zarza et al. 2008; Chan et al. 2012; Fontanella et al. 2012; Olave et al. 2015; Werneck et al. 2015; Román-Palacios et al. 2018; Bernardo et al. 2019; Camurugi et al. 2022; Carvalho et al. 2024; Rogers et al. 2024). However, as in most vertebrate groups, these estimates are often based on a limited number of species, typically at shallow evolutionary scales, and frequently rely on a small set of mitochondrial genes.

To address this issue, we integrated recently sequenced mitochondrial data with existing mitogenomic data to conduct comprehensive phylogenetic analyses, assessing evolutionary rate variation among Pleurodonta mitochondrial genes. Specifically, we analyzed their mitochondrial genomes to estimate its mitochondrial substitution rates. Using fossil-calibrated Bayesian phylogenetic analyses, we inferred molecular evolutionary rates across several families and characterized new nearly complete mitogenomes for seven *Tropidurus* species: *T. guarani*, *T. melanopleurus*, *T.* sp.* nov.* (species currently under formal description), *T. spinulosus*, *T. tarara*, *T. teyumirim*, and *T. xanthochilus*. We expect that these newly estimated rates will improve the precision of molecular clock dating and evolutionary inferences in squamates, offering deeper insights into the evolutionary processes influencing biodiversity patterns in this group.

## Methods

We assembled a comprehensive dataset of Pleurodontan mitochondrial genomes available from GenBank by November 2024, including seven recently described sequences from different *Tropidurus* species (Salles et al. 2025). One Chamaleonidae species (*Calluma parsonii*) was included as an outgroup, resulting in a final dataset with 28 species (Table 1). Only coding regions (13 genes) and the two mitochondrially encoded ribosomal RNAs (12 and 16 s) were used. We excluded additional mtDNA markers because they represent regions that are either non-coding and hyper-variable (D-loop) or ultra-conserved (tRNAs), and therefore inadequate for molecular clock calibrations. We separately aligned each mitochondrial gene with MAFFT v7.471 (Katoh and Standley 2013) using specific customized settings (-globalpair, -maxiterate 1000, -adjustdirection). Alignments were broadly examined by eye, and AMAS (Borowiec 2016) was used to concatenate alignments and compute final summary statistics.

**Table 1:** Species used in all analyses in the present study

Species	Family	GenBank accession number
*Calluma parsonii*	Chamaeleonidae	AB474915
*Basiliscus vittatus*	Corytophanidae	AB218883
*Amblyrhynchus cristatus*		NC_028031
*Conolophus subcristatus*	Iguanidae	NC_028030
*Cyclura pinguis*		NC_027089
*Iguana delicatissima*		NC_044899
*Iguana iguana*		NC_002793
*Leiocephalus personatus*	Leiocephalidae	AB266739
*Liolaemus darwinii*		NC_057242
*Liolaemus millcayac*	Liolaemidae	NC_057243
*Liolaemus parthenos*		NC_057244
*Chalarodon madagascariensis*		AB266748
*Oplurus grandidieri*	Opluridae	AB218720
*Holbrookia lacerata*		NC_041001
*Phrynosoma blainvillii*	Phyrnosomatidae	NC_036492
*Sceloporus occidentalis*		AB079242
*Urosaurus nigricaudus*		NC_026308
*Anolis punctatus*		NC_044125
*Anolis cybotes*	Polychrotidae*	AB218960
*Polychrus marmoratus*		AB266749
*Plica plica*		AB218961
***Tropidurus guarani***		PZ278525, PZ278518, PZ277233, PZ317045, PZ317052, PZ317059, PZ317066, PZ317073, PZ317080, PZ317087, PZ317094, PZ317101, PZ317108, PZ317115, PZ317122
***Tropidurus melanopleurus***		PZ278529, PZ278522, PZ277237, PZ317049, PZ317056, PZ317063, PZ317070, PZ317077, PZ317084, PZ317091, PZ317098, PZ317105, PZ317112, PZ317119, PZ317126
***Tropidurus* sp.* nov***		PZ278530, PZ278523, PZ277238, PZ317050, PZ317057, PZ317064, PZ317071, PZ317078, PZ317085, PZ317092, PZ317099, PZ317106, PZ317113, PZ317120, PZ317127
***Tropidurus spinulosus***	Tropiduridae	PZ278526, PZ278519, PZ277234, PZ317046, PZ317053, PZ317060, PZ317067, PZ317074, PZ317081, PZ317088, PZ317095, PZ317102, PZ317109, PZ317116, PZ317123
***Tropidurus tarara***		PZ278527, PZ278520, PZ277235, PZ317047, PZ317054, PZ317061, PZ317068, PZ317075, PZ317082, PZ317089, PZ317096, PZ317103, PZ317110, PZ317117, PZ317124
***Tropidurus teyumirim***		PZ278531, PZ278524, PZ277239, PZ317051, PZ317058, PZ317065, PZ317072, PZ317079, PZ317086, PZ317093, PZ317100, PZ317107, PZ317114, PZ317121, PZ317128
***Tropidurus xanthochilus***		PZ278528, PZ278521, PZ277236, PZ317048, PZ317055, PZ317062, PZ317069, PZ317076, PZ317083, PZ317090, PZ317097, PZ317104, PZ317111, PZ317118, PZ317125

New mitochondrial genomes are in bold

*Traditionally, *Anolis* was classified within Polychrotidae. However, molecular phylogenetic studies have led to a major taxonomic reassessment. Recent evidence supports placing *Anolis* and related genera within Dactyloidae, rendering Polychrotidae paraphyletic or obsolete. While some taxonomic authorities now recognize Dactyloidae, references to Polychrotidae persist in the literature. Therefore, our option here was to consider *Anolis* and *Polychrus* to form a distinct phylogenetic group, despite of their taxonomical status. The group monophyly was not enforced and, hence, taxonomic arrangements had no influence in our analyses

We emphasize that reconstructing a fully resolved topology or estimating divergence times for Pleurodonta as a whole was not a primary objective of this study. Our inferences are based exclusively on mitochondrial markers, and the taxon sampling represents only a subset of pleurodontan diversity, excluding several major lineages. Accordingly, the resulting phylogenies and divergence time estimates were used as a comparative and controlled temporal framework to evaluate methodological choices (e.g., partitioning strategies, substitution model specification, and calibration effects) under realistic levels of molecular heterogeneity, rather than as a comprehensive or definitive phylogenetic hypothesis for the group. To ensure internal consistency, calibration points were restricted to nodes with broadly supported monophyly, as monophyly was enforced for all calibrated clades. As a result, some fossil calibrations commonly used in the literature were excluded when associated with groups whose phylogenetic status remains uncertain (see Table S1).

### Partitioning Schemes

We evaluated alternative partitioning strategies to assess their impact on substitution rate estimates. First, we implemented a fully partitioned scheme in which substitution rates were estimated independently for each mitochondrial gene and for each codon position within protein-coding sequences using BEAST v2.7 (Drummond and Rambaut 2007). In this framework, tree topologies were linked across partitions, whereas clock models were unlinked both among genes and among codon positions within genes. Site models were linked across codon positions within genes but unlinked between genes. Substitution model selection in this case was conducted using BEAST Model Test (bModelTest; Bouckaert and Drummond 2017) under the ‘namedExtended’ model set, allowing models to be sampled during the MCMC via reversible-jump proposals. Under this framework, substitution models are not fixed a priori for each gene. Instead, bModelTest treats the substitution model itself as a parameter and samples it during the MCMC using reversible-jump proposals across the predefined model space. As a result, divergence time and substitution rate estimates are marginalized over substitution model uncertainty rather than conditioned on a single selected site model.

To explicitly test the effect of partitioning, we also implemented a data-driven partitioning scheme inferred with ModelFinder (Kalyaanamoorthy et al. 2017), which identifies the partition configuration best supported by the data under a Maximum Likelihood framework. Estimates obtained under this scheme were compared with those from the fully partitioned approach obtained through BEAST, allowing us to assess whether model complexity influences substitution rate estimates.

### Effect of Calibration Points on Substitution Rate Estimates

We also implemented different calibration strategies to understand its possible effects on substitution rate estimates. Specifically, estimates were obtained separately from calibrated and non-calibrated analyses under both the fully partitioned and the ModelFinder-based approaches. Calibration points within the Pleurodonta clade were obtained consulting the specialized literature, prioritizing those that have been used in multiple evolutionary studies, and which are broadly supported by the fossil record (Table 2).

**Table 2:** Values (million years, MY) of uniformly distributed calibration priors applied in dating analyses, based on both fossil and molecular data

Calibrated node (MRCA prior)	Species included	Lower value	Upper value	References
Pleurodonta	All except outgroup (*Calluma parsonii*)	65	85	Conrad and Norell (2007); Townsend et al. (2011); Prates et al. (2015); Scarpetta (2019)
*Anolis*	*Anolis cybotes, Anolis punctatus*	40	60	Sherratt et al. (2015); Zheng and Wiens (2016); Román-Palacios et al. (2018)
Phrynosomatidae	*Holbrookia lacerata, Phrysonoma blainvillii, Sceloporus occidentalis, Urosaurus nigricaudus*	35	55	Townsend et al. (2011); Leaché and Linkem (2015); Zheng and Wiens (2016)
*Liolaemus* 2	*Liolaemus darwinii, Liolaemus parthenos, Liolaemus millcayac*	30	45	Portelli et al. (2022)
*Liolaemus* 1	*Liolaemus darwinii, Liolaemus parthenos*	10	25	Fontanella et al. (2012); Portelli et al. (2022)

Settings for calibration Bayesian prior mean, standard deviation and offset are provided. MRCA = most recent common ancestor. An offset of 0.5 was used in all analyses

### Bayesian Estimation of Mitochondrial Nucleotide Evolution Rates

Substitution rates were estimated in BEAST v2.7 (Drummond and Rambaut 2007) under a relaxed molecular clock with an uncorrelated log-normal distribution (*ucld*), combined with either a Yule or Calibrated Yule speciation model, depending on the analysis. The relaxed *ucld-*model assumes independent substitution rates across branches, as there is no assumed correlation between the rate of a given lineage and that of its ancestor. This model requires a prior for the mean clock rate. For coding sequences, we set the mean clock rate to 0.01 substitutions/site/MY, and for rRNAs, to 0.0055, based on prior estimates for various Pleurodonta species (Supporting Table S1). A normal distribution was used for the *ucld* mean rate prior, with the above values as the mean, a standard deviation (Sigma) of 0.005 for coding sequences and 0.0015 for rRNAs, with these same values used as the Offset. This strategy assures that a hard lower bound is imposed on the clock rate, excluding negative or biologically implausible near-zero values while preserving the overall shape of the prior distribution. Hyperparameter values and prior distributional forms were evaluated through preliminary, exploratory analyses in which alternative distributions and parameter combinations were tested to assess prior behavior, exclude biologically implausible rate values, and ensure adequate MCMC mixing and convergence. Therefore, the final prior specification reflects a balance between empirical expectations and stable model performance.

All analyses were conducted under the two partitioning schemes described above (i.e., a fully partitioned scheme obtained through BEAST and a data-driven scheme inferred using ModelFinder), allowing us to assess the influence of partitioning on substitution rate estimates. To each partitioning scheme, we also applied both calibrated and non-calibrated strategies, estimating substitution rates separately under each scenario. Finally, to provide a baseline for interpretation, in all cases we conducted prior-only analyses (i.e., sampling from the prior without data) to characterize the expected distribution of substitution rates under the specified priors. This enabled direct comparison between prior and posterior distributions, allowing us to assess the extent to which the data informed parameter estimates, as indicated by shifts in central tendency or reductions in variance relative to the priors.

Analyses consisted of two independent MCMC runs of 850 million generations each, with parameters sampled every 25,000 generations. Combining the two partitioning schemes, two calibration strategies (calibrated vs. non-calibrated), and two conditions (prior-only vs. posterior), we defined eight analytical configurations. As each analytical configuration was performed twice, this resulted in a total of 16 runs (excluding exploratory analyses). Convergence was assessed in Tracer v1.4 (Rambaut et al. 2007) by examining effective sample sizes (ESS), in particular for the most relevant parameters to the study objectives, including the prior, posterior, ucld mean clock rate, and calibration-related parameters. After assessing convergence (ESS values ≥ 200) and adequate mixing of parameters, we combined the log files from the two independent runs from each analytical configuration using LogCombiner (Rambaut and Drummond 2014), and report the final results based on the merged outputs (8 files produced from the independent 16 runs).

## Results

### Alignments and Evolutionary Models

The alignment of protein-coding sequences alone comprised 11,426 bp, while the inclusion of non-coding sequences increased the total length to 14,059 bp. In the fully partitioned approach, all coding genes exhibited multiple substitution models within the 95% highest posterior density (HPD) interval estimated through the bModelTest. Only the two rRNAs had a single best-fitting model to explain site substitution, specifically the GTR model (Supporting Table S2).

Under the data-driven partitioning scheme inferred using ModelFinder, the dataset was grouped into five partitions, each assigned a distinct substitution model. A detailed description of this partitioning scheme can be found in Supporting Table S3.

### Substitution Rates

**Fig. 1:**
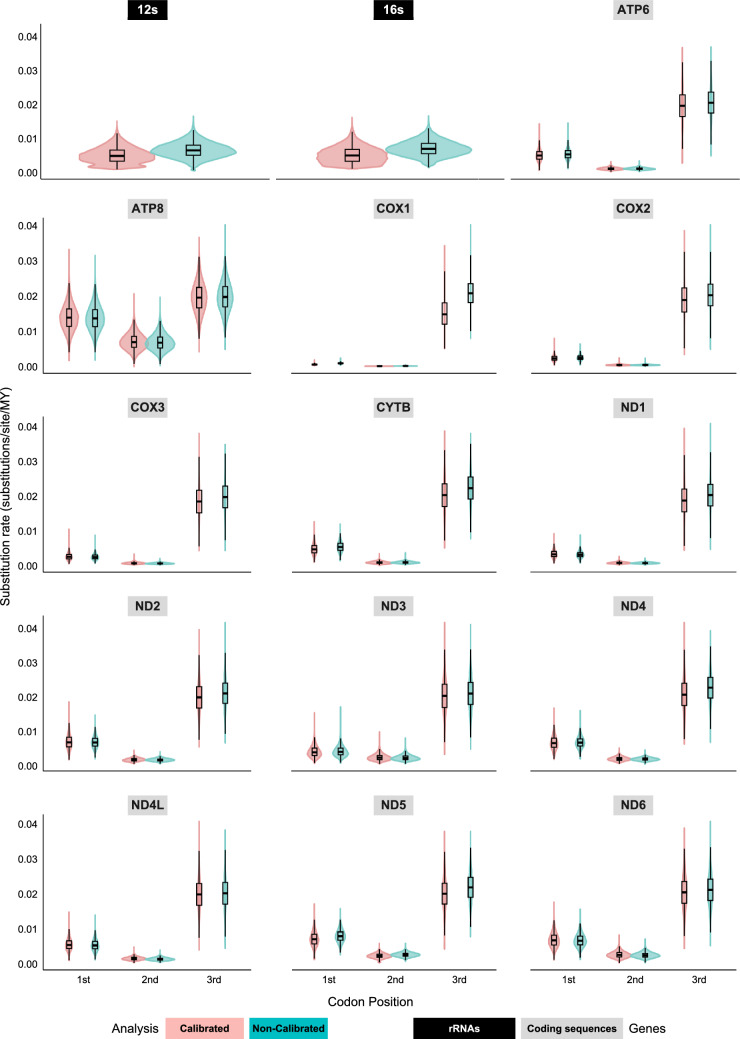
Posterior distributions of mitochondrial substitution rates (substitutions/site/MY) from calibrated (pink) and non-calibrated (blue) analyses across mitochondrial genes. Each panel represents a gene, with protein-coding genes further partitioned by codon position (1st, 2nd, and 3rd), while rRNAs are shown as single partitions. Violin plots illustrate the distribution density of posterior estimates, with embedded boxplots indicating median values and interquartile ranges

Analysis of substitution rates across codon positions revealed pronounced heterogeneity in evolutionary rates across the mitochondrial genome of Pleurodontans. For clarity, Fig. 1 and the results presented below specifically refer to the BEAST fully partitioned approach, whereas the results under the ModelFinder-based approach are provided in the Supporting Material (Figure S1 and Table S4). Importantly, the main patterns, such as the relationship between prior and posterior distributions, the fact that 95% HPD intervals do not overlap zero, and the comparison between calibrated and non-calibrated estimates were mostly consistent across both approaches, unless explicitly stated otherwise. Naturally, differences between approaches were primarily observed in the absolute values of parameter estimates for individual partitions.

When considering only protein-coding sequences, substitution rates showed a clear and consistent structure across codon positions. Third codon positions exhibited the highest rates, followed by first positions, while second positions showed substantially lower rates; this pattern was consistent across all analyzed genes (Table 3). Despite this codon-level heterogeneity, substitution rates were relatively homogeneous across genes within each codon position, as indicated by the substantial overlap of 95% HPD intervals (Table 3). In all cases, 95% HPD intervals do not overlap zero, supporting parameter identifiability and indicating that estimates were informed by the data. At the gene level, ND2, ND4, and ND5 consistently showed higher substitution rates, especially at third codon positions, whereas COX1, COX2, and COX3 exhibited lower rates across all positions. In contrast to coding regions, non-coding regions evolved approximately one order of magnitude more slowly (Fig. 1; Table 3). Substitution rates in rRNA genes (12 and 16S) were also substantially lower than those of third codon positions, but broadly comparable to first positions and, in several cases, higher than those of second positions. Finally, calibrated analyses generally produced lower substitution rate estimates than non-calibrated ones. However, the substantial overlap in 95% HPD intervals indicates no strong evidence for systematic differences between the two strategies (Table 3).

**Table 3:** Nucleotide substitution rates per site per million years estimated from 13 mitochondrial protein-coding genes (3rd codon position) and 2 rRNAs across 27 Pleurodontan species plus one outgroup

Gene	Non-calibrated			Calibrated		
	ucld mean rate	Stdev	95% HPD	ucld mean rate	Stdev	95% HPD
12s	0.00653	0.00222	0.00234–0.01100	0.00488	0.00235	0.00100–0.00926
16s	0.00703	0.00219	0.00294–0.01160	0.00515	0.00237	0.00121–0.00944
ATP6_1	0.00538	0.00160	0.00242–0.00860	0.00505	0.00169	0.00208–0.00854
ATP6_2	0.00104	0.00031	0.00047–0.00168	0.00104	0.00035	0.00043–0.00179
ATP6_3	0.02051	0.00460	0.01140–0.02950	0.01955	0.00473	0.01020–0.02870
ATP8_1	0.01410	0.00323	0.00795–0.02050	0.01430	0.00329	0.00792–0.02090
ATP8_2	0.00799	0.00209	0.00405–0.01220	0.00816	0.00211	0.00416–0.01240
ATP8_3	0.01954	0.00383	0.01200–0.02710	0.01934	0.00387	0.01170–0.02690
COX1_1	0.00087	0.00020	0.00051–0.00130	0.00056	0.00019	0.00026–0.00092
COX1_2	0.00011	0.00003	0.00005–0.00018	0.00007	0.00003	0.00002–0.00013
COX1_3	0.02093	0.00401	0.01310–0.02880	0.01536	0.00430	0.00775–0.02370
COX2_1	0.00248	0.00072	0.00119–0.00398	0.00292	0.00079	0.00085–0.00388
COX2_2	0.00044	0.00015	0.00018–0.00074	0.00041	0.00015	0.00013–0.00073
COX2_3	0.01980	0.00442	0.01140–0.02870	0.01815	0.00484	0.00843–0.02750
COX3_1	0.00249	0.00081	0.00104–0.00413	0.00262	0.00098	0.00084–0.00453
COX3_2	0.00061	0.00020	0.00025–0.00102	0.00066	0.00026	0.00021–0.00116
COX3_3	0.01970	0.00456	0.01060–0.02840	0.01848	0.00473	0.00922–0.02760
CYTB_1	0.00520	0.00135	0.00268–0.00791	0.00461	0.00150	0.00189–0.00768
CYTB_2	0.00096	0.00029	0.00043–0.00154	0.00091	0.00031	0.00034–0.00155
CYTB_3	0.02118	0.00439	0.01280–0.03000	0.01920	0.00455	0.01020–0.02800
ND1_1	0.00314	0.00088	0.00154–0.00495	0.00329	0.00113	0.00126–0.00560
ND1_2	0.00073	0.00021	0.00034–0.00117	0.00078	0.00027	0.00029–0.00134
ND1_3	0.02000	0.00452	0.01130–0.02890	0.01853	0.00477	0.00920–0.02800
ND2_1	0.00686	0.00166	0.00371–0.01010	0.00694	0.00215	0.00289–0.01120
ND2_2	0.00163	0.00040	0.00088–0.00245	0.00169	0.00052	0.00071–0.00273
ND2_3	0.02118	0.00429	0.01300–0.02960	0.01985	0.00458	0.01060–0.02880
ND3_1	0.00389	0.00143	0.00140–0.00674	0.00376	0.00159	0.00102–0.00694
ND3_2	0.00205	0.00078	0.00074–0.00362	0.00214	0.00039	0.00057–0.00402
ND3_3	0.02021	0.00459	0.01130–0.02920	0.01940	0.00491	0.00958–0.02900
ND4_1	0.00655	0.00148	0.00372–0.00949	0.00647	0.00190	0.00283–0.01010
ND4_2	0.00193	0.00045	0.00107–0.00283	0.00197	0.00058	0.00085–0.00309
ND4_3	0.02175	0.00423	0.01350–0.03000	0.01977	0.00461	0.01060–0.02870
ND4L_1	0.00541	0.00162	0.00248–0.008673	0.00550	0.00169	0.00237–0.00889
ND4L_2	0.00133	0.00041	0.00059–0.002150	0.00150	0.00049	0.00061–0.00248
ND4L_3	0.02024	0.00450	0.01120–0.028800	0.01981	0.00459	0.01080–0.02890
ND5_1	0.00826	0.00179	0.004775–0.01180	0.00736	0.00216	0.00332–0.01200
ND5_2	0.00282	0.00060	0.001702–0.00408	0.00254	0.00073	0.00113–0.00411
ND5_3	0.02228	0.00424	0.014200–0.03090	0.02030	0.00465	0.01130–0.03010
ND6_1	0.00700	0.00179	0.00358–0.01050	0.00715	0.00208	0.00333–0.01130
ND6_2	0.00284	0.00077	0.00142–0.00437	0.00299	0.00091	0.00132–0.00483
ND6_3	0.02128	0.00440	0.01290–0.03000	0.02052	0.00415	0.01160–0.02920

These rates were inferred using BEAST with a relaxed clock model assuming a lognormal distribution. The reported values represent the combined results from two independent runs (see Methods for details).

Comparisons between prior and posterior distributions indicated consistent posterior updating across most parameters, with shifts in central tendency, limited overlap between distributions, and/or reduced variance relative to the priors (Supporting FiguresS2–S6). In any case, some exceptions were observed: under the fully partitioned scheme, prior and posterior distributions showed greater overlap for rRNA genes compared to coding sequences, suggesting comparatively lower information content in these partitions. At the codon-position level, first-position partitions generally showed clear prior-posterior divergence, except for ATP8, where this pattern was less pronounced. In contrast, all second-position partitions exhibited consistent and well-defined differences between prior and posterior distributions. Third-position partitions showed the greatest degree of overlap, although still with noticeable posterior updating, including reduced variance and shifts in central tendency. In the ModelFinder-based approach, prior-posterior overlap was generally more pronounced than in the fully partitioned approach, particularly in the non-calibrated analyses, indicating comparatively weaker parameter resolution under this strategy (Supporting Figure S2). Despite this, posterior updating was still noticeable, especially in the calibrated analyses, where posterior distributions consistently showed reduced variance, along with shifts in central tendency.

## Discussion

### The Influence of Different Partitioning Schemes on the Estimation of Substitution Rates

Our data support the widely accepted view that codon positions evolve under distinct evolutionary dynamics (Kimura 1980). Within genes, first codon positions tend to resemble other first positions more closely than second or third positions, with the same logic applying to each codon class (Bofkin and Goldman 2007), a pattern clearly recovered in our analyses (Fig. 1). Third codon positions exhibited the highest substitution rates, with markedly greater heterogeneity relative to first and second positions.

This pattern is best understood in light of the genetic code. Any substitution at the second codon position, for example, results in a nonsynonymous change, rendering these sites subject to strong purifying selection. Consequently, most second-position sites evolve slowly, whereas a smaller fraction (likely experiencing relaxed functional constraints) can accumulate substitutions more rapidly (Bofkin and Goldman 2007). This asymmetry generates the skewed rate distributions observed here and reinforces that heterogeneity among codon positions reflects biologically grounded differences in selective regimes rather than statistical noise. Studies drawing inferences from mitochondrial coding data must therefore explicitly accommodate these structured differences, as ignoring them may introduce phylogenetic artifacts (Hassanin 2006).

Direct methodological implications can also be drawn from these findings. First, that partitioned analyses that explicitly model position-specific rate variation might be essential for accurately representing mitochondrial sequence evolution. Codon-position models, in particular, may provide a more realistic framework by accommodating distinct evolutionary dynamics among site classes. Consistent with this, substitution model testing (both under the fully partitioned scheme and the ModelFinder-based approach) also revealed substantial heterogeneity in the best-fitting models across mitochondrial genes and partitions, often favoring parameter-rich formulations that incorporate variation in substitution rates and nucleotide frequencies (Tables S2-S3). Overly simplistic models may, therefore, incorrectly estimate evolutionary parameters by failing to account for multiple substitutions at the same site (Yang and Nielsen 2000; Anisimova and Kosiol 2009; Duchêne et al. 2014), potentially propagating bias into phylogenetic inference (Buckley et al. 2001; Su et al. 2014).

Importantly, under a Bayesian framework such as the bModelTest approach adopted here (Bouckaert and Drummond 2017), the occurrence of multiple substitution models within the 95% HPD interval for a given gene reflects posterior uncertainty under model averaging. In this approach, models are sampled during the MCMC rather than fixed a priori, and their posterior support represents the relative probability of alternative model configurations given the data. Accordingly, this pattern should not be interpreted as conflicting model assignments, but as an explicit quantification of model uncertainty. By contrast, model-selection approaches such as ModelFinder (Kalyaanamoorthy et al. 2017) identify a single best-fitting model under a given criterion. While both strategies are valid, they reflect different inferential philosophies: model averaging propagates model uncertainty into downstream estimates, whereas fixed-model approaches condition inference on specific selected models. Careful consideration of these alternatives is important, as model specification can influence the precision and robustness of evolutionary inferences, including divergence-time estimation and substitution rate calibration.

### Difference Between Coding and Non-Coding Regions

Mitogenomes are often regarded as more reliable than single-gene approaches for divergence time estimation, as the latter frequently overestimate node ages (e.g., Duchêne et al. 2011). In this context, our analyses show that substitution rate variation across mitochondrial coding regions in Pleurodontan squamates is structured primarily by codon position, with comparatively smaller differences among genes, with comparatively smaller differences among genes. Although the mitogenome evolves as a single non-recombining unit and generally conveys a consistent phylogenetic signal, we still detected subtle but consistent differences among genes within each codon class. Importantly, these differences were modest in magnitude, with substantial overlap in 95% HPD intervals among genes, indicating that gene-specific effects, while biologically meaningful, are comparatively less pronounced than codon-level variation. These results align with previous evidence that evolutionary pressures differ among mitochondrial genes (Saccone et al. 1999; Xu et al. 2006). For example, under the fully partitioned approach, ND2, ND4, and ND5 exhibited higher substitution rates, whereas COX1, COX2, and COX3 were consistently among the most conserved (Table 3). This pattern indicates that, even if underlying mutation processes are broadly similar across the mitochondrial genome, differences in fixation probabilities imposed by the genetic code are sufficient to generate measurable rate variation.

From a methodological perspective, this hierarchical structure of rate variation suggests that much of the heterogeneity can be captured by explicitly accounting for codon position, while gene-level differences provide a secondary layer of refinement. Consequently, carefully selected subsets of coding genes, particularly when analyzed under appropriate partitioning schemes, may approximate broader mitochondrial rate patterns while reducing sequencing effort and computational burden. In this context, prioritizing loci with intermediate substitution rates and strong phylogenetic signal may represent an efficient strategy. At the same time, locus-specific properties remain important for study design and interpretation. For instance, 12 and 16S rRNA genes require particular attention, as they exhibit substitution rates substantially lower than third codon positions and broadly comparable to first codon positions (Table 3), likely reflecting functional constraints on ribosome assembly and saturation in conserved regions (Mueller 2006; Duchêne et al. 2011). More generally, loci with limited variability, high homoplasy, or alignment ambiguity should be critically evaluated to avoid reducing analytical resolution (Zardoya and Meyer 1996; Non et al. 2007). Defining appropriate gene subsets therefore requires taxon-specific substitution models and robust calibration strategies, underscoring the importance of tailored analytical frameworks, an approach facilitated by the partition-specific estimates presented here.

In sum, our results also caution against the expectation that a single pooled substitution rate across all mitochondrial genes should serve as a universal reference value. Even if intergenic differences are relatively modest within codon classes, variation persists among codon positions, reflecting fundamental differences in selective constraints imposed by the genetic code, and also between coding and non-coding regions. Consequently, summarizing all coding regions into a single estimate necessarily conflates distinct evolutionary processes and may obscure biologically meaningful patterns or introduce bias into divergence-time estimation. The differences in estimates observed between the approaches adopted here (fully partitioned and ModelFinder-based) further reinforce this point, highlighting that substitution rate estimates are sensitive to the chosen partitioning scheme. Consequently, failure to account for this structured heterogeneity may lead to systematic misestimation of substitution rates, which can propagate into biased divergence time inferences and distort interpretations of evolutionary tempo and mode.

### Substitution Rates Heterogeneity Depending on the Presence of Calibration Points

Our study also evaluated the influence of temporal calibrations on mitochondrial substitution rate estimates in Pleurodonta. Calibrated analyses broadly yielded lower substitution rate estimates, often with reduced variance, compared to non-calibrated ones (Fig. 1 and Table 3, but also see the Supporting Material), consistent with previous findings that well-constrained calibrations can reduce biases in molecular rate estimation (Hipsley and Müller 2014; Warnock et al. 2015). This highlights the importance of integrating multiple, well-justified fossil constraints (particularly at deeper nodes) to improve the temporal scaling of phylogenetic inferences in groups with complex evolutionary histories, such as Pleurodonta (Blankers et al. 2013; Alencar et al. 2024).

However, despite the general tendency for calibrated analyses to yield lower substitution rate estimates, the substantial overlap in 95% HPD intervals between calibrated and non-calibrated results indicates that there is no strong evidence for systematic differences between the two approaches (Table 3). A plausible explanation for this pattern is that our dataset already contained a strong signal for relative rate variation, particularly under the employed partitioned relaxed-clock framework. Because rate heterogeneity is explicitly modeled through partitioning and the relaxed-clock framework, the inclusion of fossil constraints possibly introduced limited adjustments to substitution rate estimates. In practice, this resulted in small shifts in central tendency and minor variance, while the overall distribution of rates across genes and codon positions remained mostly unchanged. Accordingly, the differences observed between calibrated and non-calibrated analyses are best interpreted as minor adjustments to the same underlying signal, rather than as evidence for distinct substitution rate dynamics.

### Balancing Model Complexity and Parameter Resolution in Bayesian Analyses

In Bayesian phylogenetic inference, increasing model complexity does not necessarily imply overparameterization in the classical sense, as parameters are estimated jointly and regularized through prior distributions and hierarchical model structure (Yang 2005). This is particularly relevant in partitioned analyses, where different data subsets (e.g., genes or codon positions) are allowed to evolve under distinct parameter sets. While such schemes better capture known biological heterogeneity, they also increase model dimensionality and raise questions about parameter identifiability and the extent to which estimates are informed by the data. This balance is further shaped by the structure of relaxed-clock models. Under the *ucld* model, branch-specific rates are not estimated as independent parameters but are instead drawn from a shared distribution (Drummond and Rambaut 2007). This hierarchical formulation constrains the parameter space and mitigates overfitting, partially offsetting the increase in complexity introduced by partitioning. At the same time, relaxed-clock models provide a more realistic representation of molecular evolution than strict-clock approaches, which assume a single rate across all lineages and may lead to biased divergence-time estimates when rate heterogeneity is present (Duchêne et al. 2014; Membrebe et al. 2019).

Given this context, a key question is whether increased model complexity compromises parameter identifiability or leads to estimates that are weakly informed by the data. In our analyses, most parameters showed prior-posterior divergence, indicating effective updating and demonstrating that the data were informative for substitution rate estimation (Supporting Material). In these cases, posterior distributions were primarily shaped by the likelihood rather than reflecting prior assumptions alone. However, this pattern was not uniform across all partitions. For the rRNA genes (12 and 16S), prior and posterior distributions exhibited substantial overlap, indicating comparatively lower information content. More generally, stronger prior-posterior overlaps like this likely reflects reduced parameter identifiability, where the likelihood surface is relatively flat and the available data provide limited constraint on parameter estimates. This pattern may arise either from limited data or from intrinsic biological properties, such as low substitution rates and strong functional constraints, which reduce the number of informative substitutions and limit evolutionary variance.

In this context, our results highlight a central trade-off in Bayesian analyses. Increasing model complexity through additional partitions does not necessarily improve inferential resolution, as some partitions may remain weakly informed and partially dependent on prior assumptions. As a result, the benefits of increased biological realism must be balanced against the statistical identifiability of parameters. Several strategies may help address this limitation in future studies. Increasing taxon sampling may enhance the amount of phylogenetic signal available per partition, while the inclusion of additional and more informative calibration points can improve the temporal scaling of rate estimates. From a modeling perspective, partitioning strategies may be optimized by merging poorly informed partitions or by applying model selection approaches that explicitly balance model fit and complexity. Our results further show that model selection frameworks such as ModelFinder (Kalyaanamoorthy et al. 2017) provide a useful empirical benchmark for evaluating whether alternative partitioning schemes achieve an appropriate balance between model complexity and data support. In our case, the ModelFinder-based scheme captured broader patterns of rate variation but did so at the cost of reduced parameter resolution relative to the fully partitioned approach. Notably, this reduction in resolution was less pronounced in calibrated analyses, where posterior distributions showed clearer departures from the priors, including reduced variance and narrower HPD intervals. More flexible representations of sequence evolution may also improve model fit, albeit at increased computational cost.

### One Rate to Rule Them All?

The partitioning scheme for estimating and reporting substitution rates (e.g., by gene, codon position, or site) should be guided by the characteristics of the dataset and the specific objectives of the study. Different partitions capture distinct evolutionary dynamics: more conserved regions tend to retain reliable phylogenetic signal over deeper timescales, whereas less constrained sites (such as third codon positions), due to their elevated substitution rates, are typically more informative for recent divergences and for assessing rate variation among genes. In this study, we provide substitution rate estimates for all mitochondrial genes and codon positions (Table 3), as well as partition-specific estimates inferred under the ModelFinder-based approach (Supporting Material). We hope this framework will allow researchers to select the set of rates that best matches the scope, assumptions, and temporal scale of their analyses when empirically applying our estimated rates to their studies.

When partition-specific rates are available, their use should be explicitly aligned with the phylogenetic depth of the question being addressed. Faster-evolving partitions may be particularly informative for recent divergences and demographic inference, whereas more conserved regions are generally more reliable for deeper timescales, where substitution saturation becomes a concern. Ignoring these differences may reduce resolution at shallow timescales or introduce noise and saturation effects at deeper nodes, ultimately limiting the reliability of evolutionary inference. As previously discussed, rather than supporting the use of a single genome-wide mitochondrial rate, our results favor a partition-aware and question-driven framework for substitution rate selection. In this context, some approaches extend this logic further by estimating substitution rates at the level of individual sites (e.g., Meyer and von Haeseler 2003; Puller et al. 2020). Importantly, while biologically realistic, such strategies may involve substantial computational costs and will introduce model complexity.

### Pleurodontan Evolutionary Dynamics and Future Perspectives

The substitution rate estimates from this study (up to 0.02 substitutions/site/MY, approximately) align with prior estimates reported for Pleurodontan lineages (Supporting Table S1).Notably, the commonly used rates we used as references closely match those estimated here for third codon positions, as well as for analyses in which partitions are combined (e.g., under the ModelFinder-based approach), suggesting that widely adopted values may primarily reflect signals driven by faster-evolving sites or aggregated partition schemes.

The close agreement of our substitution rate estimates with those previously reported for Pleurodonta also highlights the relative stability of mitochondrial evolutionary rates within the group. Prior research has demonstrated that mitochondrial substitution rates tend to cluster within narrow ranges among closely related taxa, often reflecting shared evolutionary constraints (Päckert et al. 2007; Pons et al. 2010). On the other hand, while mitochondrial protein-coding genes show conserved rate variation patterns across vertebrates, a phenomenon stable for ~ 450 million years (Broughton and Reneau 2006), the drivers of this variation remain poorly understood. This gap highlights an opportunity to explore how structural, functional, and selective pressures differentially shape mitochondrial gene evolution (Caron and Domingos 2026).

Conversely, we note that many of earlier estimated for the group values were extrapolated from studies of distantly related taxa rather than empirically derived from lineage-specific calibrations. This reinforces the reliability of our methodological framework, which incorporated different partitioning schemes, appropriate substitution models, and strong prior calibration strategies. Furthermore, this also reinforces that our chosen priors, which were informed by values for different taxa within the Pleurodontan clade already reported on the literature (Table 2), proved to be robust.

Importantly, while our analyses provide robust substitution rate estimates for Pleurodonta as a whole, offering a useful baseline for future molecular dating studies, the use of lineage-specific rates may be more appropriate depending on the taxonomic scope and objectives of a given study. Estimating rates at finer phylogenetic scales can capture variation associated with ecological, physiological, or demographic factors known to influence mitochondrial evolution (e.g., Welch et al. 2008; Nabholz et al. 2016; Jing et al. 2024), potentially refining evolutionary inferences within Pleurodonta. In this context, branch-specific rate estimates, such as those implemented in PAML (Yang 2007), represent a widely used framework for quantifying lineage-level rate variation, although their reliability depends on factors such as sequence quality, alignment accuracy, and model specification (Rasmussen and Kellis 2007; Yan et al. 2023).

However, the application of lineage-specific approaches remains constrained by data availability. The still limited number of complete mitochondrial genomes across several pleurodontan lineages restricts the feasibility of more comprehensive, lineage-resolved analyses. In addition, such approaches can be computationally demanding, as they require the specification of tailored models that account for codon position, partition-specific dynamics, and rate heterogeneity across the mitochondrial genome, as implemented here. Their accuracy further depends on well-supported calibration points, which remain limited for many taxa. Incomplete sampling or poorly justified calibrations may introduce substantial uncertainty into divergence-time estimates (Zheng and Wiens 2015; Schenk 2016).

Furthermore, beyond the framework adopted here, several alternative approaches for estimating substitution rates are available. For example, germline-based estimates (e.g., Bergeron et al. 2023) are particularly informative for characterizing average nuclear genomic variation, but require multi-generational genomic data and remain taxonomically restricted (Chintalapati and Moorjani 2020; Bergeron et al. 2023).

## Conclusion

In this study we examined the phylogenetic utility of nearly complete mitogenomes regarding the estimation of substitution rates, offering critical insights into the application of mitochondrial data in evolutionary studies. Despite the study’s focus on a specific taxonomic scope (the Pleurodonta clade), the framework applied here may be broadly applicable across different taxa and divergence times. Our findings reveal relatively homogeneity in substitution rates across Pleurodontan mitochondrial protein-coding genes, but heterogeneity between these and non-coding regions. As a consequence of that, while subsets of informative genes might approximate these results, their effectiveness depends on robust methodological frameworks and careful taxon-specific selection. Future research will be essential to determine whether this heterogeneity arises primarily from conserved replication mechanisms that drive variation in mutation rates across genomic regions, the effects of natural selection on individual genes, a combination of these factors, or other evolutionary processes.

There is also a considerable amount of difference in substitution rates when accounting for codon positions. Although this heterogeneity is structured and largely predictable, our results show that explicitly accounting for it through partition-informed rate estimates improves the accuracy of evolutionary inferences. Accordingly, the choice of modeling and partitioning schemes represents a critical step in analyses based on mitochondrial markers and should be carefully evaluated. Furthermore, our results indicate that model complexity should be evaluated in terms of parameter identifiability and data informativeness, rather than parameter count alone, and support the use of partitioned relaxed-clock models as a biologically realistic framework for mitochondrial rate estimation. In this context, the mitogenome-wide and partition-specific estimates presented here provide a practical and flexible reference framework that can be refined as additional data become available, while their application should be balanced against data availability, model complexity, and potential sources of uncertainty.

Finally, our results provide a valuable reference for future investigations into evolutionary dynamics specifically within the Pleurodonta clade and its closely related lineages, offering a foundation for comparative studies across Squamata. We then hope that our findings establish a foundation for optimizing mitochondrial phylogenetics in squamates, facilitating more accurate evolutionary reconstructions across diverse taxa and timescales.

## Supplementary Information

Below is the link to the electronic supplementary material.

Supplementary file1 (DOCX 1231 KB)

## Data Availability

The data underlying this article, including phylogenetic datasets, corresponding trees, input and output files for all analyses, and any other relevant supplementary files are available in Zenodo, at 10.5281/zenodo.15952175.

## Declarations

### Generative AI in Scientific Writing

During the preparation of this work the authors used ChatGPT to review their own original translation of the text from Portuguese to English. After using this tool/service, both authors reviewed and edited the content as needed and take full responsibility for the content of the published article.
