# Supplementary material for: Mitochondrial Genome Evolution: The Influence of Partitioning, Calibration, and Gene Heterogeneity on Pleurodontan Substitution Rates
Source: J Mol Evol. 2026 May 30;94(3):573–87. doi: 10.1007/s00239-026-10324-5 (PMC13294174; doi:10.1007/s00239-026-10324-5)
Supplement: Supplementary file 1 — Supplementary file1 (DOCX 1231 KB) [file 239_2026_10324_MOESM1_ESM.docx]

**Mitochondrial genome evolution: the influence of partitioning, calibration, and gene heterogeneity on Pleurodontan substitution rates**

Matheus M. A. Salles¹^*^, Fabricius M. C. B. Domingos¹

¹Departamento de Zoologia, Universidade Federal do Paraná, Curitiba/PR, Brazil

^*^Author for correspondence: Matheus Salles, Programa de Pós-Graduação em Zoologia, Universidade Federal do Paraná (Departamento de Zoologia, Setor de Ciências Biológicas, Centro Politécnico, Avenida Cel. Francisco H Santos, Jardim das Américas, 81531-980, Curitiba/PR, Brasil). E-mail: matheusmaciel.salles@gmail.com

**Table S1**. References regarding values for substitution rate priors adopted in the present study.

| Gene | Values | References |
| --- | --- | --- |
| COX1 | 0.01 | Phrynosomatidae: Bernardo et al., 2019;  *Tropidurus:* Camurugi et al., 2022 |
| CYTB | 0.019355 \| 0.0113 \| 0.0223 | *Cyclura*: Rogers et al., 2024; Liolaemidae*:* Olave et al., 2015; *Liolaemus:* Fontanella et al., 2012; *Tropidurus:* Werneck et al., 2015; |
| ND1 | 0.013876 | *Oplurus:* Chan et al., 2012 |
| ND2 | 0.013 | *Anolis:* Román-Palacios et al., 2018 |
| ND4 | 0.0113 \| 0.0078 | *Cyclura*: Rogers et al., 2024; Iguaninae: Zarza et al., 2008 |
| 12s & 16s | 0.006339 \| 0.00576 | Liolaemidae*:* Olave et al., 2015; *Liolaemus:* Fontanella et al., 2012; *Tropidurus:* Carvalho et al., 2024; |

**Table S2.** Model selection results from BEAST under the fully partitioned approach, including only models within the 95% HPD interval for each gene. For a detailed description of partition-specific models, see the full documentation available on [Zenodo](https://doi.org/10.5281/zenodo.19458031). The four-digit model code represents how substitution rates are grouped, following the order of relative rates for A-C, A-G, A-T, C-G, C-T, and G-T. A complete list of model codes can be found here: <https://taming-the-beast.org/tutorials/Substitution-model-averaging/>.

| Gene | Non-calibrated | Calibrated |
| --- | --- | --- |
|  | Models | |
| 12s | SYM/GTR | SYM/GTR |
| 16s | SYM/GTR | SYM/GTR |
| ATP6 | K80/HKY, _123324_, _123345_, _123425_/TVM, SYM/GTR | K80/HKY, _121131_/TN93, _123324_, _123345_, _123425_/TVM, SYM/GTR |
| ATP8 | K80/HKY, _121131_/TN93_, 123321_/K81_,_  _123324, 123341_/TIM_, 123345_ | K80/HKY, _121131_/TN93, _123321_/K81, _123324_,  _123341_/TIM, _123345_, _123345_ /TVM |
| COX1 | _121131_/TN93, _123341_/TIM_, 123345_ | _121131_/TN93, _123341_/TIM, _123345_ |
| COX2 | K80/HKY, _121131_/TN93_, 123321_/K81_,_  _123324, 123341_/TIM | K80/HKY, _121131_/TN93, _123321_/K81,  _123324_, _123341_/TIM |
| COX3 | K80/HKY, _121131_/TN93, _123321_/K81, _123324, 123345_ | K80/HKY, _121131_/TN93, _123321_/K81_,_  _123324_, _123341_/TIM |
| CYTB | _123345_ /TVM, SYM/GTR | _123324_, _123345_ /TVM, SYM/GTR |
| ND1 | _123324_, _123345_, _123425_/TVM, SYM/GTR | _123324_, _123345_, _123345_ /TVM, SYM/GTR |
| ND2 | _123324_, _123345_, _123425_/TVM | _123324_, _123324_, _123345_ /TVM |
| ND3 | K80/HKY, _121131_/TN93, _123321_/K81, _123341_/TIM, _123345_ | K80/HKY, _121131_/TN93_, 123321_/K81,  _123324_, _123341_/TIM |
| ND4 | _123345_, SYM/GTR | _123345_, SYM/GTR |
| ND4L | K80/HKY, _123321_/K81, _123324_,  _123341_/TIM, _123345_, _123425_/TVM, SYM/GTR | K80/HKY, _121131_/TN93_, 123321_/K81, _123324_,  _123341_/TIM, _123345, 123345_ /TVM |
| ND5 | _123324_, _123345_, _123425_/TVM | _123324_, _123345_, _123345_ /TVM |
| ND6 | _123425_/TVM, SYM/GTR | _123345_ /TVM, SYM/GTR |

**Table S3.** Model selection results from ModelFinder.

| Substitution model | Partition scheme |
| --- | --- |
| GTR+F+I+G4 | 12s + 16s + ATP6_1 + ATP8_1 + CYTB_1 + ND1_1 + ND2_1 + ND3_1 + ND4_1 + ND4L_1 + ND5_1 |
| TVM+F+I+G4 | ATP6_2 + ATP8_2 + COX1_2 + COX2_2 + COX3_2 + CYTB_2 + ND1_2 + ND2_2 + ND3_2 + ND4_2 + ND4L_2 + ND5_2 |
| TIM3+F+I+G4 | ATP6_3 + ATP8_3 + COX1_3 + COX2_3 + COX3_3 + CYTB_3 + ND1_3 + ND2_3 + ND3_3 + ND4_3 + ND4L_3 + ND5_3 |
| SYM+I+G4 | COX1_1 + COX2_1 + COX3_1 + ND6_1 + ND6_2 |
| TPM3+F+I+G4 | ND6_3 |

**Table S4.** Nucleotide substitution rates (substitutions/site/MY) estimated for five mitochondrial partitions defined under the ModelFinder-based partitioning scheme, across 27 Pleurodontan species plus one outgroup. Partitions comprise: (#1) 12S and 16S rRNAs plus first codon positions of ATP6, ATP8, CYTB, ND1–ND5; (#2) second codon positions of ATP6, ATP8, COX1–COX3, CYTB, ND1–ND5; (#3) third codon positions of ATP6, ATP8, COX1–COX3, CYTB, ND1–ND5; (#4) first codon positions of COX1–COX3 and ND6 plus second codon positions of ND6; and (#5) third codon positions of ND6. Estimates are shown for both non-calibrated and calibrated analyses and include the mean ucld rate, standard deviation, and 95% highest posterior density (HPD) intervals. All rates were inferred in BEAST under a relaxed molecular clock with a normal prior on the mean rate. Reported values correspond to the combined results of two independent MCMC runs (see Methods for details).

| **Partition** | **Non-calibrated** | | | **Calibrated** | | |
| --- | --- | --- | --- | --- | --- | --- |
|  | ucld mean rate | Stdev | 95% HPD | ucld mean rate | Stdev | 95% HPD |
| #1 | 0.01561 | 0.00458 | 0.00692 – 0.02470 | 0.01296 | 0.00518 | 0.00297 – 0.02240 |
| #2 | 0.01342 | 0.00517 | 0.00309 – 0.02310 | 0.01204 | 0.00594 | 0.00054 – 0.02160 |
| #3 | 0.01914 | 0.00453 | 0.01060 – 0.02820 | 0.01460 | 0.00328 | 0.00865 – 0.02120 |
| #4 | 0.01371 | 0.00510 | 0.00348 – 0.02320 | 0.01278 | 0.00536 | 0.00143 – 0.02230 |
| #5 | 0.01365 | 0.00507 | 0.00364 – 0.02380 | 0.01287 | 0.00523 | 0.00234 – 0.02210 |


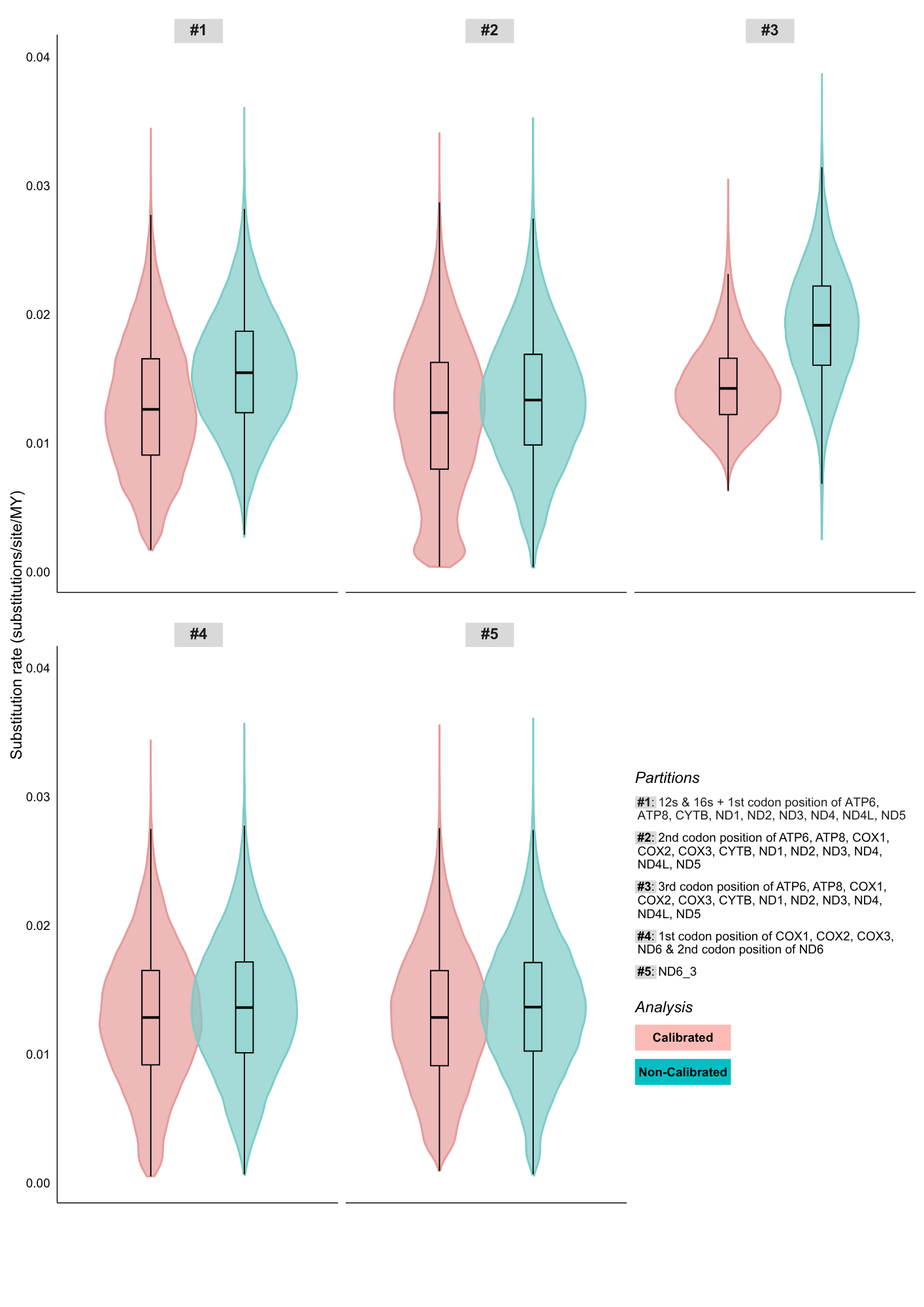


**Fig. S1.** Posterior distributions of mitochondrial substitution rates (substitutions/site/MY) from calibrated (pink) and non-calibrated (blue) analyses across mitochondrial partitions (each panel represents a different partition obtained through ModelFinder analyses). Violin plots illustrate the distribution density of posterior estimates, with embedded boxplots indicating median values and interquartile ranges.


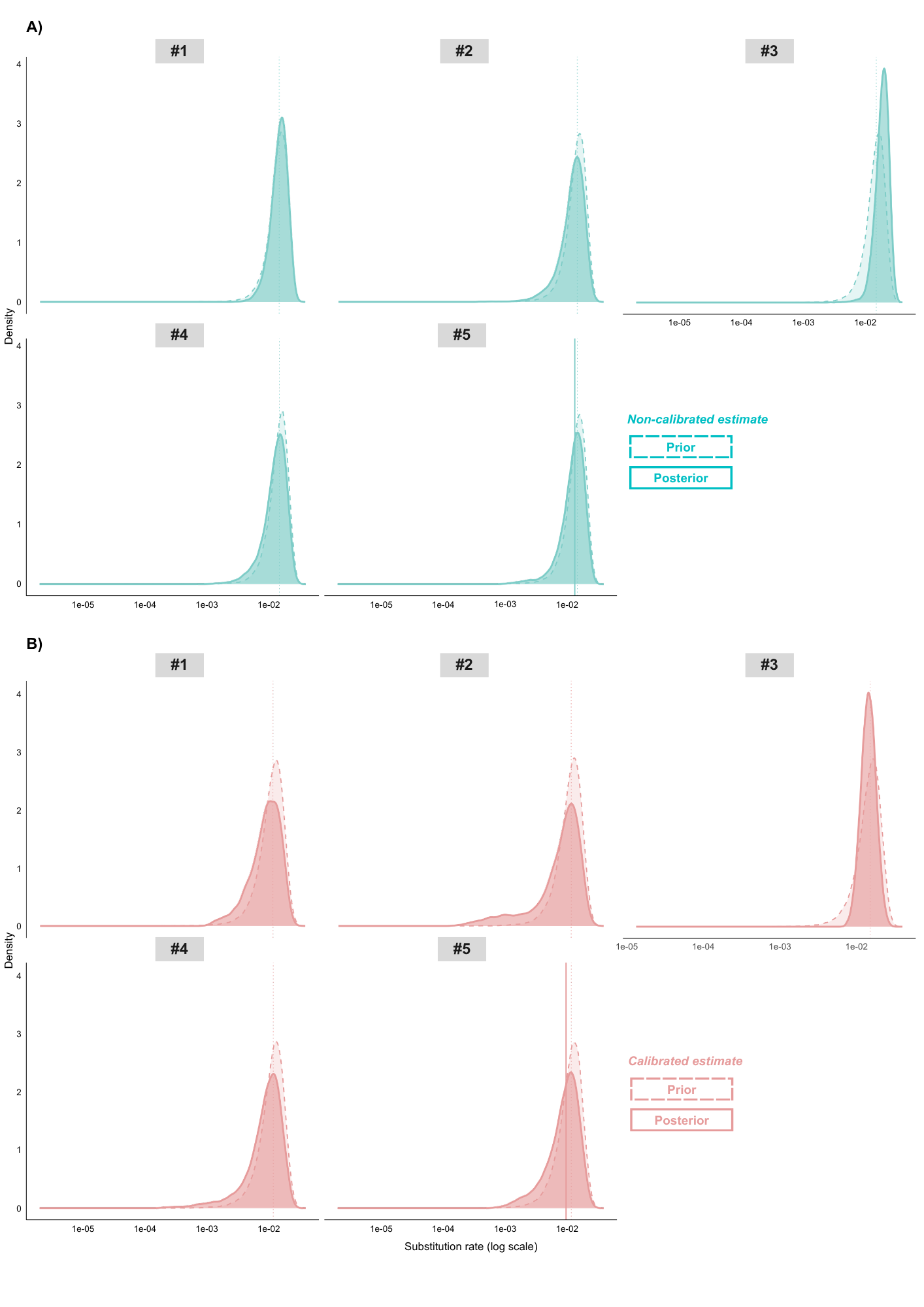


**Fig. S2.** Prior and posterior distributions of substitution rates (substitutions/site/MY, log scale) for five mitochondrial partitions defined under the ModelFinder-based scheme. Partitions comprise: (#1) 12S and 16S rRNAs plus first codon positions of ATP6, ATP8, CYTB, ND1–ND5; (#2) second codon positions of ATP6, ATP8, COX1–COX3, CYTB, ND1–ND5; (#3) third codon positions of ATP6, ATP8, COX1–COX3, CYTB, ND1–ND5; (#4) first codon positions of COX1–COX3 and ND6 plus second codon positions of ND6; and (#5) third codon positions of ND6. Panels show comparisons between prior (dashed) and posterior (solid) distributions for (A) non-calibrated and (B) calibrated analyses. Vertical lines indicate median estimates.


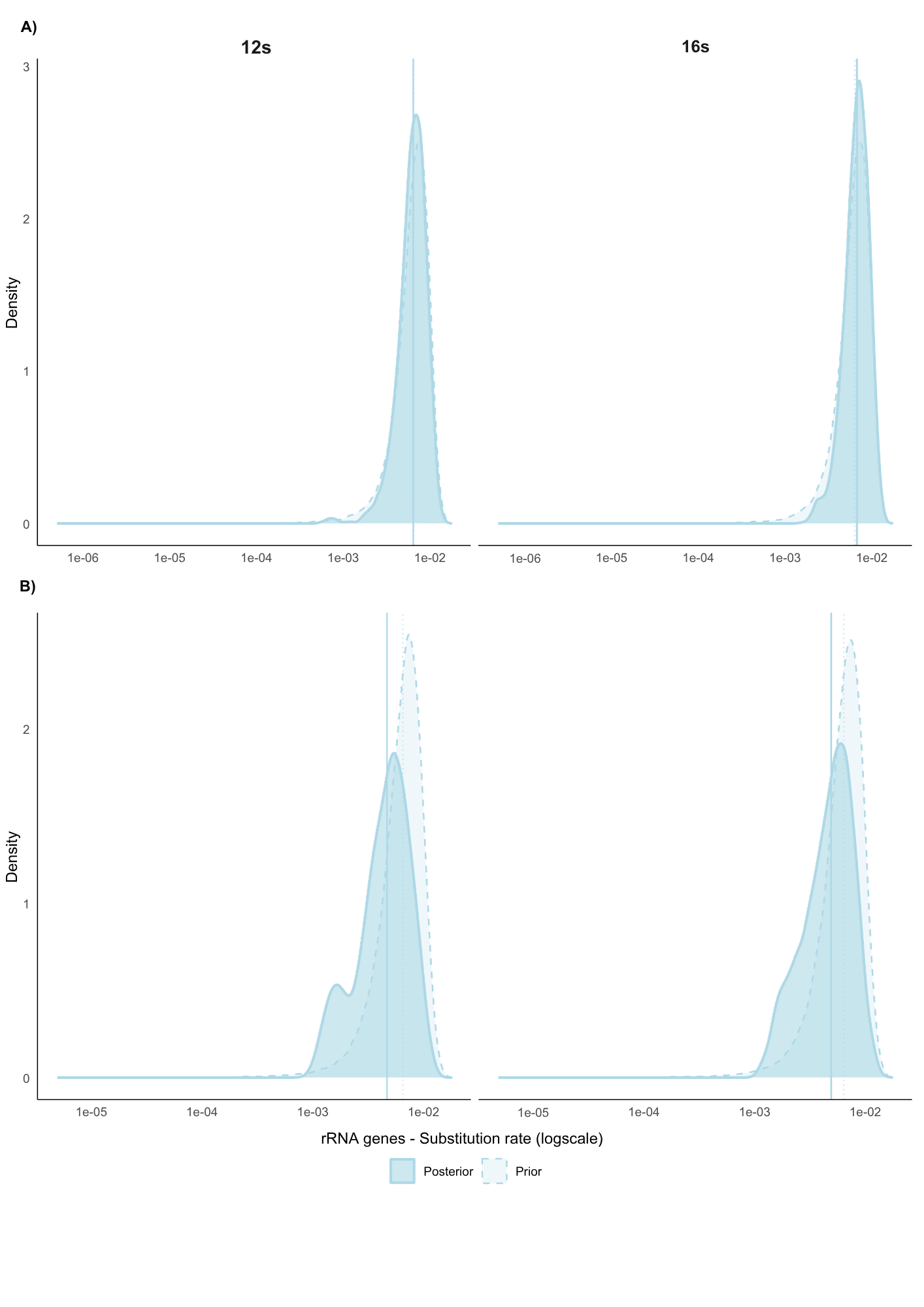


**Fig. S3.** Prior and posterior distributions of substitution rates (substitutions/site/MY, log scale) for mitochondrial rRNA genes (12S and 16S). Panels show comparisons between prior (gray, dashed) and posterior (blue) distributions. (A) Non-calibrated analyses; (B) calibrated analyses. Vertical lines indicate median estimates.


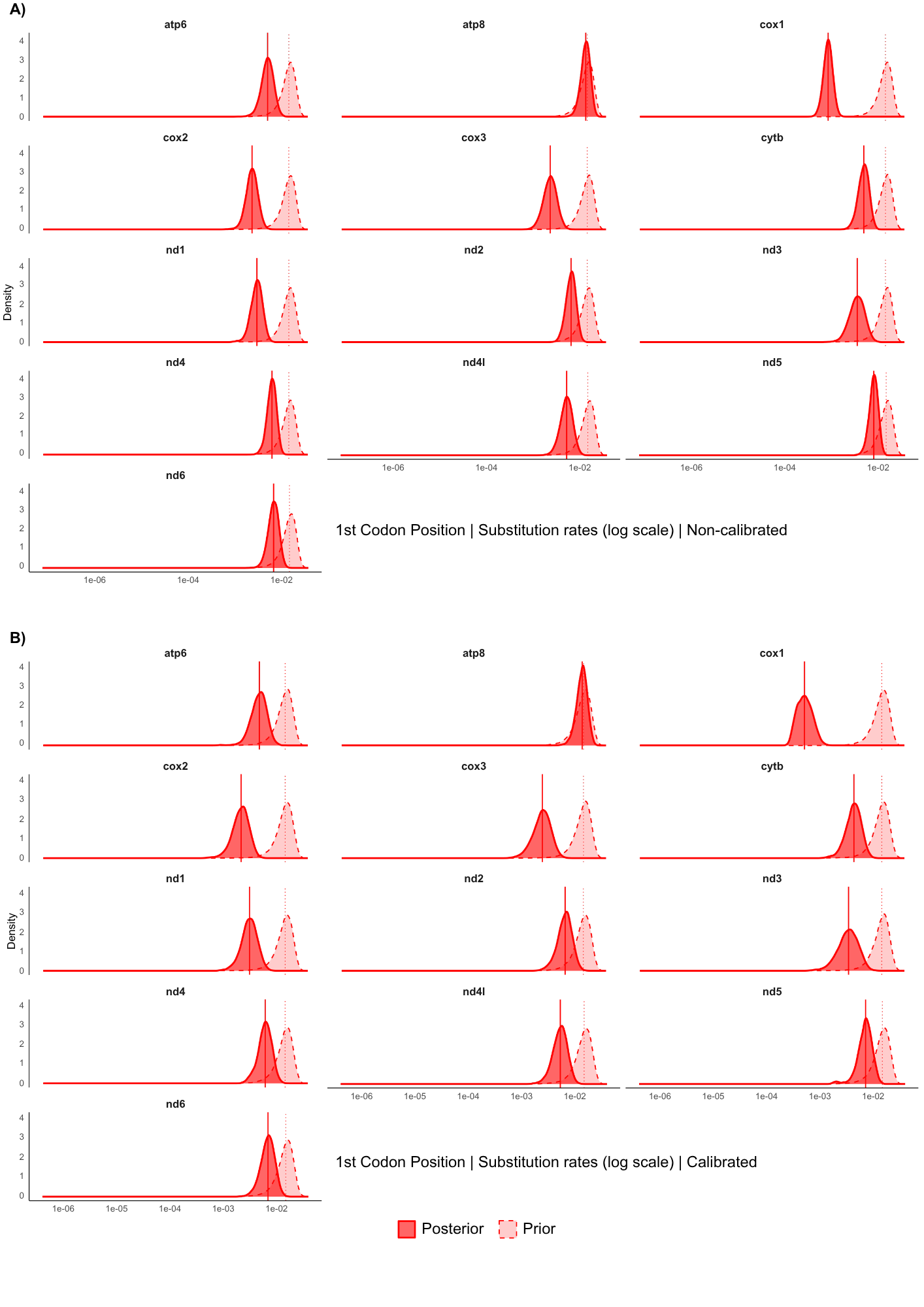


**Fig. S4**. Prior and posterior distributions of substitution rates (substitutions/site/MY, log scale) for first codon positions across mitochondrial protein-coding genes. Each panel corresponds to a gene, comparing posterior (solid red) and prior (dashed red) distributions. (A) Non-calibrated analyses; (B) calibrated analyses. Vertical lines indicate median estimates.


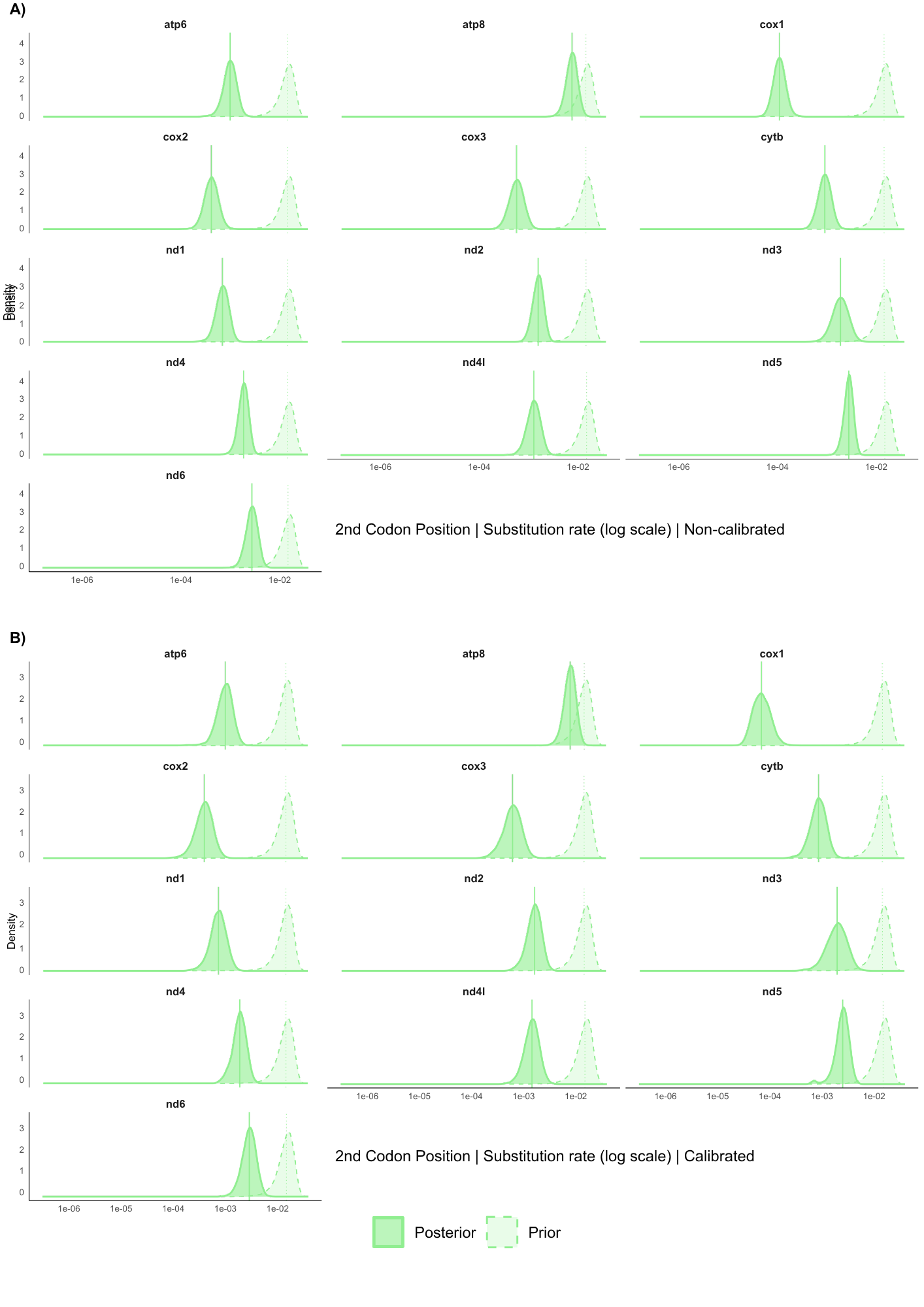


**Fig. S5**. Prior and posterior distributions of substitution rates (substitutions/site/MY, log scale) for second codon positions across mitochondrial protein-coding genes. Each panel corresponds to a gene, comparing posterior (solid red) and prior (dashed red) distributions. (A) Non-calibrated analyses; (B) calibrated analyses. Vertical lines indicate median estimates.


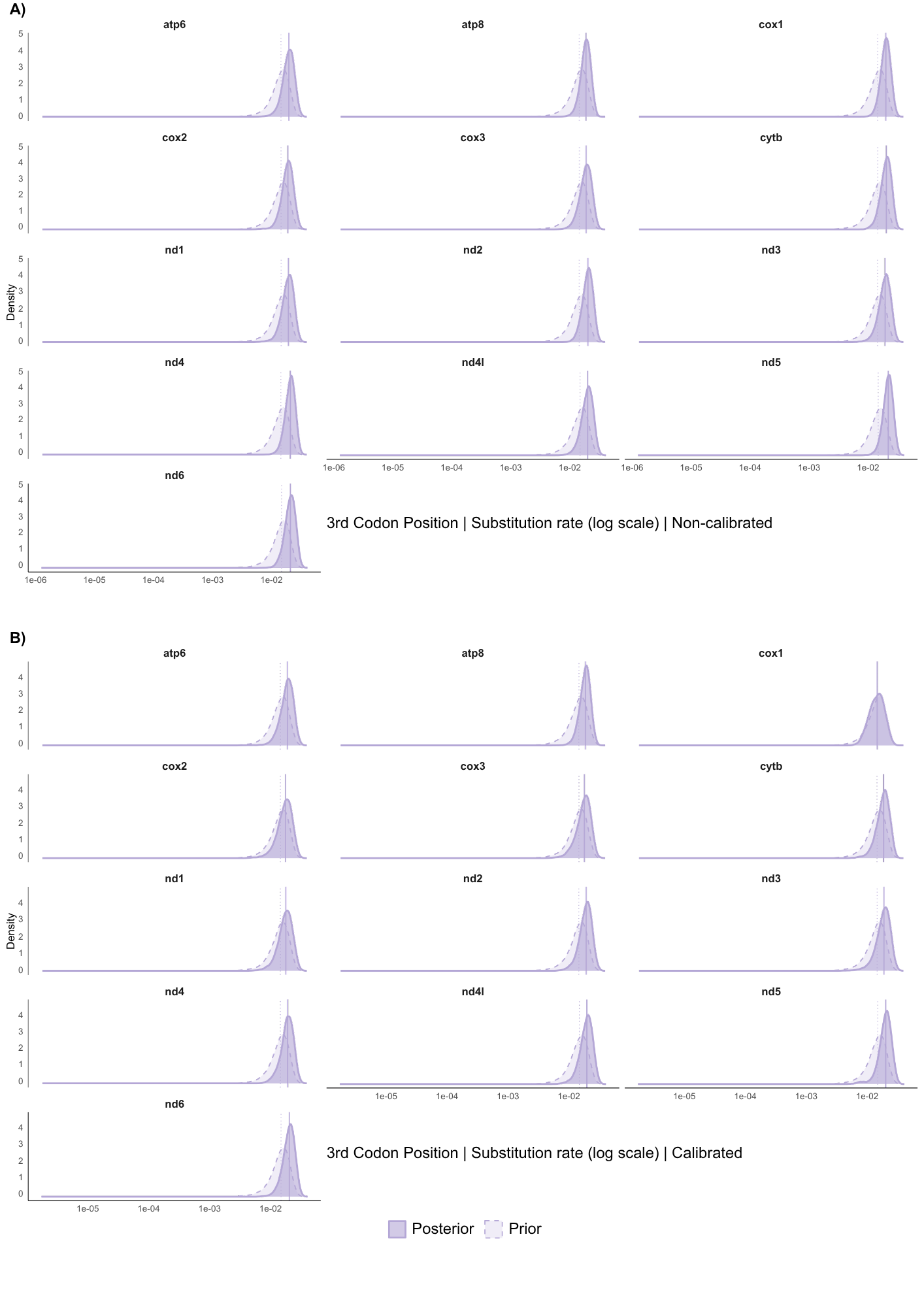


**Fig. S6.** Prior and posterior distributions of substitution rates (substitutions/site/MY, log scale) for third codon positions across mitochondrial protein-coding genes. Each panel corresponds to a gene, comparing posterior (solid red) and prior (dashed red) distributions. (A) Non-calibrated analyses; (B) calibrated analyses. Vertical lines indicate median estimates.
